# Examining the effect of family-based intervention programs used in psychosis on the disease: a systematic review

**DOI:** 10.1192/j.eurpsy.2024.604

**Published:** 2024-08-27

**Authors:** Z. Kutlutürk, M. C. Aktaş, C. H. Ayhan

**Affiliations:** Mental Health and Psychiatric Nursing, Van Yuzuncu Yil University, Van, Türkiye

## Abstract

**Introduction:**

Psychosis is a complex mental health condition that can have a significant impact on individuals and their families. Family-based intervention programs have been developed to provide support and education to both individuals with psychosis and their families. These programs aim to improve clinical and family outcomes, reduce relapse rates, and enhance the overall well-being of individuals and their families . This study examined the effect of family-based intervention programs used in psychosis on the disease by synthesizing evidence from a systematic review of relevant studies.Family interventions have shown promising results in improving clinical and family outcomes in long-standing psychosis (Sadath et al., 2015). These interventions focus on improving relationships through problem-solving and enhancing the understanding of the illness and its treatment (Kuipers et al., 2010).

**Objectives:**

The aim of this study was to evaluate the effect of intervention programs implemented for the families of individuals diagnosed with psychosis on the course of the disease.

**Methods:**

The study was conducted between August and September 2023 in 3 databases (PubMed, Cochrane Library, Science Direct) using the keywords “psychosis”, “family interventions”, “family in psychosis”. These databases were preferred because they contain a significant amount of evidence-based literature in the field of biomedical sciences and psychology. Studies conducted between 2013 and 2023, whose full texts were accessed and written in Turkish and English were included in the study.

**Results:**

As of September 2023, 16 national and international research articles on the subject have been reached and the literature review continues. When the literature review is finalized, all study results will be presented together.

**Conclusions:**

This review provides an overview of the effects of intervention programs implemented for the families of individuals diagnosed with psychosis on the course of the disease and solution suggestions. Family-based intervention programs have shown promise in improving clinical and family outcomes in psychosis. These programs focus on enhancing relationships, providing education, and reducing relapse rates. However, the implementation of family interventions in routine clinical services can be challenging due to various barriers. Peer support programs have emerged as a valuable addition to family interventions, providing a supportive environment for families to share their experiences and support one another. Future research should focus on addressing barriers to implementation and further exploring the benefits of peer-driven family support services in early intervention programs for psychosis.

**Disclosure of Interest:**

None Declared

